# Supplementary material for: A UMLS-based spell checker for natural language processing in vaccine safety
Source: BMC Med Inform Decis Mak. 2007 Feb 12;7:3. doi: 10.1186/1472-6947-7-3 (PMC1805499; doi:10.1186/1472-6947-7-3)
Supplement: Additional file 13 — RAP application directory. Sets up directory for RAP (RDF application for PHP) source code [file 1472-6947-7-3-S13.gz › rap/doc/tests/Test_Manipulate_MemModel.htm]

Test Manipulate RDF Model


### RDF API for PHP V0.9.1

# Test: Manipulate MemModel

This example is part of the RAP - Rdf API for PHP documentation.

Used document: example1.rdf

`<?xml version="1.0"?>  
<rdf:RDF   
xml:base="http://www.w3.org/Home/Lassila.rdf#"  
xmlns:rdf="http://www.w3.org/1999/02/22-rdf-syntax-ns#"  
xmlns:dc="http://purl.org/dc/elements/1.1/"  
xmlns:ex="http://example.org/stuff/1.0/"  
xmlns:s="http://description.org/schema/">  
<rdf:Description rdf:about="http://www.w3.org/Home/Lassila">  
<s:Creator>  
<rdf:Description rdf:nodeID="b85740">  
<rdf:type rdf:resource="http://description.org/schema/Person"/>  
<ex:Name rdf:datatype="http://www.w3.org/TR/xmlschema-2#string">Ora
Lassila</ex:Name>  
<ex:Email>lassila@w3.org</ex:Email>  
</rdf:Description>  
</s:Creator>  
</rdf:Description>  
</rdf:RDF>`

  
  

### 1. Parse and show model

`// Include RAP  
define("RDFAPI_INCLUDE_DIR", "./../api/");  
include(RDFAPI_INCLUDE_DIR . "RDFAPI.php");`

`// Filename of an RDF document  
$base="example1.rdf";`

`// Create a new MemModel   
$model = ModelFactory::getDefaultModel();`

`// Load and parse document  
$model->load($base);`

`// Output model as HTML table  
$model->writeAsHtmlTable();`

**Output:**

|  |  |  |  |
| --- | --- | --- | --- |
| **Base URI:** http://www.w3.org/Home/Lassila.rdf# | | | **Size:** 4 |
| **No.** | **Subject** | **Predicate** | **Object** |
| 1. | Resource: http://www.w3.org/Home/Lassila | Resource: http://description.org/schema/Creator | Blank Node: b85740 |
| 2. | Blank Node: b85740 | RDF Node: http://www.w3.org/1999/02/22-rdf-syntax-ns#type | Resource: http://description.org/schema/Person |
| 3. | Blank Node: b85740 | Resource: http://example.org/stuff/1.0/Name | Literal: Ora Lassila **(rdf:datatype="http://www.w3.org/TR/xmlschema-2#string")** |
| 4. | Blank Node: b85740 | Resource: http://example.org/stuff/1.0/Email | Literal: lassila@w3.org |

  
  

### 2. Add some statements

`// Ceate new statements and add them to model  
$statement1 = new Statement(new Resource("http://www.w3.org/Home/Lassila"),  
new Resource("http://description.org/schema/Description"),  
new Literal("Lassila's personal Homepage", "en"));  
  
$statement2 = new Statement(new Resource("http://www.w3.org/Home/Lassila"),  
new Resource("http://description.org/schema/Description"),  
new Literal("Lassilas persönliche Homepage ", "de"));`

`$model->add($statement1);  
$model->add($statement2);`

`$model->writeAsHtmlTable();`

**Output:**

|  |  |  |  |
| --- | --- | --- | --- |
| **Base URI:** http://www.w3.org/Home/Lassila.rdf# | | | **Size:** 6 |
| **No.** | **Subject** | **Predicate** | **Object** |
| 1. | Resource: http://www.w3.org/Home/Lassila | Resource: http://description.org/schema/Creator | Blank Node: b85740 |
| 2. | Blank Node: b85740 | RDF Node: http://www.w3.org/1999/02/22-rdf-syntax-ns#type | Resource: http://description.org/schema/Person |
| 3. | Blank Node: b85740 | Resource: http://example.org/stuff/1.0/Name | Literal: Ora Lassila **(rdf:datatype="http://www.w3.org/TR/xmlschema-2#string")** |
| 4. | Blank Node: b85740 | Resource: http://example.org/stuff/1.0/Email | Literal: lassila@w3.org |
| 5. | Resource: http://www.w3.org/Home/Lassila | Resource: http://description.org/schema/Description | Literal: Lassila's personal Homepage **(xml:lang="en")** |
| 6. | Resource: http://www.w3.org/Home/Lassila | Resource: http://description.org/schema/Description | Literal: Lassilas pers�nliche Homepage **(xml:lang="de")** |

  
  

### 3. Search Statements

`// Build search index to speed up searches.  
$model->index();`

`// Search model 1  
$homepage = new Resource("http://www.w3.org/Home/Lassila");  
$res = $model->find($homepage, NULL, NULL);`

`$res->writeAsHtmlTable();  
echo "<P>";`

**Output:**

|  |  |  |  |
| --- | --- | --- | --- |
| **Base URI:** http://www.w3.org/Home/Lassila.rdf# | | | **Size:** 3 |
| **No.** | **Subject** | **Predicate** | **Object** |
| 1. | Resource: http://www.w3.org/Home/Lassila | Resource: http://description.org/schema/Creator | Blank Node: b85740 |
| 2. | Resource: http://www.w3.org/Home/Lassila | Resource: http://description.org/schema/Description | Literal: Lassila's personal Homepage **(xml:lang="en")** |
| 3. | Resource: http://www.w3.org/Home/Lassila | Resource: http://description.org/schema/Description | Literal: Lassilas pers�nliche Homepage **(xml:lang="de")** |

### 4. Search First Matching Statement

`// Search model 2  
$description = new Resource("http://description.org/schema/Description");  
$statement = $model->findFirstMatchingStatement($homepage, $description,
NULL);`

`// Check if something was found and output result  
if ($statement) {  
echo $statement->toString();  
} else {  
echo "Sorry, I didn't find anything.";  
}  
echo "<P>";`

**Output:**

Triple(Resource("http://www.w3.org/Home/Lassila"), Resource("http://description.org/schema/Description"),
Literal("Lassila's personal Homepage", lang="en"))

  

### 5. Search Vocabulary

`// Search model 3  
$res3 = $model->findVocabulary("http://example.org/stuff/1.0/");  
$res3->writeAsHtmlTable();  
echo "<P>";`

**Output:**

|  |  |  |  |
| --- | --- | --- | --- |
| **Base URI:** http://www.w3.org/Home/Lassila.rdf# | | | **Size:** 2 |
| **No.** | **Subject** | **Predicate** | **Object** |
| 1. | Blank Node: b85740 | Resource: http://example.org/stuff/1.0/Name | Literal: Ora Lassila **(rdf:datatype="http://www.w3.org/TR/xmlschema-2#string")** |
| 2. | Blank Node: b85740 | Resource: http://example.org/stuff/1.0/Email | Literal: lassila@w3.org |

### 5. Serialize MemModel to RDF

`// Write model as RDF  
$model->writeAsHtml();`

**Output:**

<?xml version='1.0' encoding='UTF-8'?>

### 6. Save manipulated MemModel to file

`// Save model to file  
$model->saveAs("Output.rdf");`

**Output:**

File: Output.rdf
